# Neurotoxicity of the Parkinson Disease-Associated Pesticide Ziram Is Synuclein-Dependent in Zebrafish Embryos

**DOI:** 10.1289/EHP141

**Published:** 2016-06-15

**Authors:** Aaron Lulla, Lisa Barnhill, Gal Bitan, Magdalena I. Ivanova, Binh Nguyen, Kelley O’Donnell, Mark C. Stahl, Chase Yamashiro, Frank-Gerrit Klärner, Thomas Schrader, Alvaro Sagasti, Jeff M. Bronstein

**Affiliations:** 1 Department of Neurology, University of Los Angeles (UCLA), Los Angeles, California, USA; 2 Brain Research Institute, and; 3 Molecular Biology Institute, UCLA, Los Angeles, California, USA; 4 Department of Chemistry and Biochemistry, UCLA, Los Angeles, California, USA; 5 UCLA-DOE Institute, UCLA, Los Angeles, California, USA; 6 Department of Molecular, Cell, and Developmental Biology, UCLA, Los Angeles, California, USA; 7 Institute of Organic Chemistry, University of Duisburg-Essen, Essen, Germany; 8 Parkinson’s Disease Research, Education, and Clinical Center, Greater Los Angeles Veterans Affairs Medical Center, Los Angeles, California, USA

## Abstract

**Background:**

Exposure to the commonly used dithiocarbamate (DTC) pesticides is associated with an increased risk of developing Parkinson disease (PD), although the mechanisms by which they exert their toxicity are not completely understood.

**Objective:**

We studied the mechanisms of ziram’s (a DTC fungicide) neurotoxicity in vivo.

**Methods:**

Zebrafish (ZF) embryos were utilized to determine ziram’s effects on behavior, neuronal toxicity, and the role of synuclein in its toxicity.

**Results:**

Nanomolar-range concentrations of ziram caused selective loss of dopaminergic (DA) neurons and impaired swimming behavior. Because ziram increases α-synuclein (α-syn) concentrations in rat primary neuronal cultures, we investigated the effect of ziram on ZF γ-synuclein 1 (γ1). ZF express 3 synuclein isoforms, and ZF γ1 appears to be the closest functional homologue to α-syn. We found that recombinant ZF γ1 formed fibrils in vitro, and overexpression of ZF γ1 in ZF embryos led to the formation of neuronal aggregates and neurotoxicity in a manner similar to that of α-syn. Importantly, knockdown of ZF γ1 with morpholinos and disruption of oligomers with the molecular tweezer CLR01 prevented ziram’s DA toxicity.

**Conclusions:**

These data show that ziram is selectively toxic to DA neurons in vivo, and this toxicity is synuclein-dependent. These findings have important implications for understanding the mechanisms by which pesticides may cause PD.

**Citation:**

Lulla A, Barnhill L, Bitan G, Ivanova MI, Nguyen B, O’Donnell K, Stahl MC, Yamashiro C, Klärner FG, Schrader T, Sagasti A, Bronstein JM. 2016. Neurotoxicity of the Parkinson disease-associated pesticide ziram is synuclein-dependent in zebrafish embryos. Environ Health Perspect 124:1766–1775; http://dx.doi.org/10.1289/EHP141

## Introduction

Parkinson disease (PD) is a neurodegenerative disease that affects millions of individuals worldwide (Dorsey et al. 2007). Although significant progress has been made in understanding the pathophysiology of PD, the etiology remains poorly understood. There does not appear to be one simple cause of PD; rather, it likely develops from complex genetic and environmental interactions.

Several lines of evidence have implicated the involvement of α-synuclein (α-syn) in the pathogenesis of PD. The identification of mutations in the α-syn gene (*SNCA*) led to the finding that α-syn is the major component of Lewy bodies (the pathological hallmark of PD) not only in these patients but also in sporadic PD (Baba et al. 1998; Chartier-Harlin et al. 2004; Farrer et al. 2004; Ibáñez et al. 2004; Krüger et al. 1998; Nishioka et al. 2006; Polymeropoulos et al. 1997; Singleton et al. 2003; Spillantini et al. 1997). Additionally, over-expression of wild-type (WT) α-syn by gene multiplication causes fairly typical PD (Hope et al. 2004). Furthermore, people who carry an allele in the α-syn promoter (REP1 263) that confers a higher level of expression are at greater risk of developing PD (Maraganore et al. 2006). Thus, increased expression of α-syn increases the risk of incurring PD, and if expression is doubled or more, the disease will develop. REP1 263 is also associated with faster progression of PD, adding further support to the concept that increased levels of α-syn are central to the pathogenesis of PD (Ritz et al. 2012). Evidence is building that α-syn expression may also interact with environmental factors to increase the risk of developing the disease. For example, the REP1 263 allele influences susceptibility to damage from paraquat exposure and head trauma (Gatto et al. 2010; Goldman et al. 2012).

The genetics of PD have been extensively studied, but genetic susceptibility only accounts for a small percentage of patients with PD, suggesting that environmental factors play an important role (International Parkinson Disease Genomics Consortium et al. 2011; Trinh and Farrer 2013). Epidemiological studies have indicated that pesticide exposure is associated with an increased risk of developing PD (Brown et al. 2006; Goldman 2014), and a causal role for this association is supported by cell-based and animal studies (Betarbet et al. 2000; Chou et al. 2008; Fitzmaurice et al. 2013; McCormack et al. 2002; Ryan et al. 2013; Thiruchelvam et al. 2000; Wang et al. 2006). Interestingly, the mechanisms by which pesticides increase disease risk appear to be diverse. Proposed mechanisms include mitochondrial inhibition, oxidative stress, impaired protein degradation, and aldehyde dehydrogenase (ALDH) inhibition (Barnhill and Bronstein 2014; Goldman 2014).

One pesticide of particular interest is the dithiocarbamate (DTC) fungicide ziram. Residential and workplace exposure to ziram is associated with a 300% increased risk for developing PD and as much as a 600% increased risk for early-onset cases (Wang et al. 2011). Additionally, *in vitro* and *in vivo* exposure studies have shown that ziram is toxic to dopaminergic (DA) neurons in rat primary neuronal cultures (Chou et al. 2008). The mechanism of ziram’s DA toxicity appears to be related, at least in part, to its ability to impair protein degradation by inhibiting E1 ligase of the ubiquitin proteasome system (UPS) and causing accumulation of α-syn (Chou et al. 2008).

Despite the progress that has been made in understanding the mechanisms of ziram toxicity, *in vivo* studies are lacking. One reason for the scarcity of *in vivo* studies is that testing in rodent models is very expensive and time consuming. Zebrafish (*Danio rerio*, ZF) are a powerful model organism for studying the effects of environmental toxins and neurodegenerative diseases because they are rapidly developing vertebrates with a well- formed DA neuronal network similar to that of mammals (Bretaud et al. 2007; Milanese et al. 2012; Schweitzer et al. 2012; Sheng et al. 2010; Wen et al. 2008; Xi et al. 2011); they have many orthologues to human drug targets (Gunnarsson et al. 2008); and alterations in homeostasis of proteins associated with PD have been found to cause similar effects in ZF and other model organisms (Betarbet et al. 2000; Feany and Bender 2000; Prabhudesai et al. 2012). Here, we utilized ZF to further study the neurotoxicity of ziram *in vivo* and found that exposure to low concentrations led to damaged DA neurons and abnormal motor behavior. ZF do not have an orthologue to human α-syn, but ZF γ-synuclein 1 (γ1) appears to be functionally similar to α-syn (Milanese et al. 2012). ZF γ1 was found to form fibrils when over-expressed, similarly to human α-syn. Importantly, ziram’s toxicity was found to be ZF γ1-dependent.

## Methods

### Zebrafish

ZF lines (AB unless otherwise stated) were bred and maintained at 28°C in recirculating water tanks on a regulated 14 hr light/10 hr dark cycle and were fed twice a day with brine shrimp. All experiments were performed in accordance with University of California, Los Angeles (UCLA) Animal Research Committee protocols. ZF expressing green fluorescent protein (GFP) driven by the vesicular monoamine transporter promoter (VMAT2:GFP) were purchased from the UCLA core facility and were used in this study to identify VMAT2 [dopaminergic, (nor)adrenergic, serotonergic] neurons in whole embryos (Wen et al. 2008). Peripheral sensory neurons (trigeminal and Rohon-Beard) were visualized using the Tg(isl1[ss]:Gal4-VP16,UAS:EGFP)^zf154^ transgenic line, which has been referred to as Tg(sensory:GFP) (Sagasti et al. 2005).

A ZF γ1-synuclein expression construct (γ1-DsRed) composed of a T2A bicistronic configuration, driven by the *HuC* neuronal promoter and expressing monomeric DsRed, was used as previously described (Prabhudesai et al. 2012). The T2A sequence is cleaved after translation, generating two proteins from a single open reading frame and resulting in stoichiometric expression of the gene of interest and the fluorescent reporter (Tang et al. 2009). ZF embryos were injected with *HuC*-ZFγ1-T2A-DsRed or *HuC*-DsRed (control) cDNA (50 ng/μL) at the single-cell stage.

Morpholinos (MOs; Gene Tools LLC, Philomath OR) were injected (0.1 mM) at the single-cell stage for ZF γ1 (CAT​TAG​AAC​ATC​CAT​CCT​GGA​CGCT; translational blocking) or nontargeting/scrambled (CCT​CTT​ACC​TCA​GTT​ACA​ATT​TATA), as previously described (see Figure S1) (Milanese et al. 2012). The molecular tweezers CLR01 and CLR03 were prepared and purified as sodium salts, as described previously (Fokkens et al. 2005; Talbiersky et al. 2008). Specificity of the ZF γ1 MO was validated by sodium dodecyl sulfate–polyacrylamide gel electrophoresis (SDS-PAGE) (see Figure S1d).

### Zebrafish Treatments

ZF embryos, 25–35 per treatment in 10 mL of E3 media [15 mM sodium chloride (NaCl), 0.5 mM potassium chloride (KCl), 1.0 mM magnesium sulfate (MgSO_4_), 0.15 mM monopotassium phosphate (KH_2_PO_4_), 0.05 mM disodium phosphate (Na_2_HPO_4_), 1.0 mM calcium chloride (CaCl_2_), 0.7 mM sodium bicarbonate (NaHCO_3_)], were reared at 32°C and exposed to varying concentrations of ziram (98.5% purity; Chem Service, West Chester, PA). Embryos were exposed to 1 nM–1 μM [0.01% dimethyl sulfoxide (DMSO)] ziram in E3 media at 5 hr post-fertilization (hpf) or 24 hpf in a 6-well plate for 5 days for confocal microscopy and at 7 days post fertilization (dpf) for behavior. The concentrations of ziram used for the study were well below those used for spraying (approximately 8 mM) (Joyner 2015) and necessary for fungicidal activity (30 μM) (Briquet et al. 1976). VMAT2:GFP embryos were co-treated with 10 μM CLR01 (Fokkens et al. 2005; Prabhudesai et al. 2012; Sinha et al. 2011) and 50 nM ziram. A log-rank test was used for statistical analysis.

### Confocal Microscopy

VMAT2:GFP embryos (3 dpf and 5 dpf) were anesthetized using Tricane-S (Western Chemicals Inc., Ferndale, WA) at a final concentration of 50 μg/mL, then fixed in 4% paraformaldehyde (PFA) overnight and mounted in 2% low-melting agarose. Embryos were imaged using a Zeiss LSM 510 microscope at a magnification of 20×. Approximately one hundred 1.1-μm optical sections were obtained for each embryo. Section images were stacked and reconstituted into 2D and 3D images using ImageJ (Rasband 1997–2016). Neuron count analyses were conducted in a blinded manner as previously described (Fitzmaurice et al. 2013). For MO and CLR01 studies, neuron counts for treatment groups were normalized to injection and treatment controls to account for variances in fish clutches between days and for treatment and injection survival. Student’s *t*-test was used for statistical analysis.

### Histology

ZF embryos were dechorionated at 2 dpf, fixed in 4% PFA for 24 hr, immersed in 30% sucrose overnight, and cryosectioned (10 μm). For thioflavin S (ThS) staining, sections were immersed in 0.5% ThS dissolved in phosphate-buffered saline (PBS; Sigma-Aldrich, St. Louis, MO), washed with ethanol (80%), and then mounted with Vectashield+Dapi (Vector Laboratories, Burlingame, CA).

Polyclonal antibodies specific for ZF γ1 were raised using a C-terminal peptide CDFSHGGMEGGEGGE (Genscript, Piscataway, NJ). These antibodies bound specifically to ZF γ1 at the predicted mass (17 kDa) in ZF embryos and to recombinant γ1 (see Figure S1a). Furthermore, the ZF γ1 band in Western blots was markedly reduced in ZF after preabsorption with the ZF γ1 peptide used to raise the antibody (see Figure S1c). Anti-ZF γ1 antibodies did not recognize any rodent brain proteins (see Figure S1a).

For immunohistochemistry, sections were blocked in 10% normal goat serum (Jackson Labs, Bar Harbor, ME), incubated with anti-ZF γ1 antibody (1:1,000), and then incubated with an anti-rabbit Alexa Fluor 568 antibody (1:500; Life Technologies, Grand Island, NY). The sections were then mounted with Vectashield+Dapi.

### Behavioral Analysis

ZF embryos (7 dpf) were placed in 10 mL of E3 media and kept in the dark to avoid degradation of added chemicals. Drug or vehicle (5 μM apomorphine, 25 μM haloperidol, or 0.1% DMSO) were added and incubated for 30 min at 28°C before behavioral analysis. Twelve fish from each treatment group were transferred to a square 96-well plate and maintained in the dark for 10 min prior to behavioral analysis, which consisted of 10-min alternating cycles of light (100% light as specified by Zebralab, View Point, France) and dark (total 30 min in light and 30 min in the dark). Movements > 2 mm were collected every 2 min using the Zebralab system and were analyzed for distance traveled. Embryos with notochord malformations were excluded from behavioral analysis. Data were normalized to vehicle controls to account for variances between fish clutches from different days. One-way analysis of variance (ANOVA) was used for statistical analysis.

### Western and Native Blots

ZF embryos (5 dpf) were anesthetized as described above, pooled (35 embryos per treatment), de-yolked, lysed, and sonicated in either 1× SDS buffer or 1× Native PAGE sample buffer (Life Technologies). Protein concentrations were determined using the Pierce BCA protein assay (Thermo Fisher, Rockford, IL). Protein (75 μg/lane) was loaded onto SDS-PAGE or Native-PAGE gels and transferred using the XCell-II blotting system (Life Technologies). Membranes were probed with ZF γ1 1° antibody (1:2,500 dilution) or tyrosine hydroxylase TH 1° antibody (1:2,500; MAB 318; Millipore), followed by donkey anti-rabbit horseradish peroxidase (HRP) 2° antibody (1:2,500; Santa Cruz Biotechnology, Dallas, TX). Chemiluminescent substrate (Super Signal West Dura; Thermo Scientific) was used for band visualization. α-Tubulin (1:500; Sigma-Aldrich) was used as a loading control. Student’s *t*-test was used for statistical analysis.

### Expression and Purification of Zebrafish Synuclein

Competent BL21 (DE3) *Escherichia coli* bacteria were transformed with a plasmid containing the ZF synuclein gene, allowed to grow in 3 L Luria broth to 6.4 × 10^8^ cells/mL, induced with 0.5 mM isopropyl β-D-1-thiogalactopyranoside, and incubated for 3 additional hours. The bacteria were collected by centrifugation at 4,690 × *g* for 15 min and were then resuspended in 60 mL of lysis buffer containing 0.2 M Tris, 1 mM ethylenediamine tetraacetic acid (EDTA), pH 8.0, supplemented with Halt EDTA-free protease inhibitor cocktail (Thermo Scientific). The bacteria then were lysed on ice using a tip sonicator set to 3 kJ for 3 × 2 min cycles of 3 sec power on and 3 sec power off. The lysate was centrifuged at 31,920 × *g* for 20 min, and the supernatant was collected. The proteins were precipitated from the supernatant by addition of 0.23 g/mL ammonium sulfate. The solution with the ammonium sulfate was stirred on ice for 20 min and then centrifuged at 31,920 × *g* for 20 min. The supernatant was discarded, and the protein pellets were dried. The pellets were resuspended in 40 mL of 20 mM Tris, pH 8.0, and the resulting solution was dialyzed overnight against 4 L of the same buffer. The crude protein mixture was fractionated using ion-exchange Q columns (GE Healthcare, Piscataway, NJ), and a 100-mL gradient ranging from 0 to 1.0 M NaCl in 20 mM Tris, pH 8.0. The protein was purified further by size exclusion chromatography using a 2.15 × 600 mm TSKgel® G3000SW column (Tosoh Biosciences, San Francisco, CA) with elution buffer comprising 100 mM sodium sulfate, 25 mM sodium phosphate, and 1 mM sodium azide, pH 6.5. Finally, the fractions containing purified ZF synuclein were dialyzed against 10 mM sodium phosphate, pH 7.4. The purity of the protein was assessed by SDS-PAGE and Coomassie blue staining.

### Thioflavin T Fluorescence Assays

A concentrated protein solution was thawed on ice and filtered through a 0.2-μm filter before the fibril formation assay. Fibril formation assays were performed with 150-μM ZF γ1 or human α-syn as a positive control in 10 mM sodium phosphate, pH 7.4, and 10 μM thioflavin T (ThT). ThT is used for identification and staining of amyloid fibrils (Biancalana and Koide 2010). For inhibition assays with molecular tweezers, ZF γ1 was used at a concentration of 100 μM. Stock solutions were prepared by dissolving CLR01 or CLR03 at 10 mM in 10 mM sodium phosphate, pH 7.4. Solutions of ZF γ1:CLR01 were prepared in the following molar ratios: 1:0.1, 1:1, and 1:10; a solution of 1:10 ZF γ1:CLR03 was also prepared.

All assays were performed in black, Nunc 96-well optical bottom plates (Thermo Scientific). Teflon balls (0.3175 cm in diameter) were distributed into each well of the 96-well plate. Then, 200 μL of solution (four replicates per sample) was pipetted into each well. The plate was agitated at 300 rpm with a 3-mm rotation diameter in a Varioskan microplate reader (Thermo Scientific) at 37°C. Fluorescence measurements were recorded every 10–15 min at an excitation wavelength (λ_ex_) of 444 nm and an emission wavelength (λ_em_) of 482 nm, with an integration time of 200 μsec.

The ThT fluorescence signal was corrected for the background by subtracting the mean fluorescence signal acquired during the first 2 hr since the start of the assay. The fluorescence signal was normalized by dividing each measurement by the mean signal collected for the 2–4 hr during which the fluorescent reading had the largest value. If one of the sample replicates had a fluorescence reading close to the background value, its fluorescence was normalized by dividing it by the mean fluorescence reading of the remaining replicates. The data were smoothed by substituting each data point with the average value calculated from 6 data points collected before and after the point and the point itself; this is equivalent to a central moving average with a sliding 13-data-point-wide window. The normalized fluorescence signals of ZF γ1 and CLR01 (ratio 1:1) and ZF γ1 and CLR01 (1:10), and ZF γ1 and CLR03 (1:10) were calculated using the averaged fluorescence signal of maximum fluorescence acquired from the assays of ZF γ1 alone.

### Transmission Electron Microscopy

Negatively stained specimens for transmission electron microscopy (TEM) were prepared by applying 5 μL of sample onto hydrophilic, 400-mesh, carbon-coated formvar support films mounted on copper grids (Ted Pella, Inc., Redding, CA). The samples were allowed to adhere for 3 min, rinsed twice with distilled water, and stained for 1 min with 1% uranyl acetate. Grids were examined on JEM1200-EX (JEOL, Huntington Beach, CA) or T12 (FEI, Hillsboro, OR) microscopes.

### Statistical Analysis

Statistical analyses were performed using Student’s *t*-test, log-rank test, and one-way ANOVA where appropriate. A minimum significance level was set at *p* < 0.05 for all studies.

## Results

### Ziram is Toxic at Low Concentrations

ZF embryos exposed to ziram early in development resulted in marked notochord malformations (see Figure S2b,c) similarly to other dithiocarbamates (Haendel et al. 2004; Teraoka et al. 2006). To determine the toxicity of ziram without this confounder, embryos were exposed at 24 hpf, a developmental point at which the notochord is sufficiently developed. When added at 24 hpf, ziram reduced survival in a concentration-dependent manner. At 7 dpf, embryos appeared grossly normal, but survival was reduced by 80% at concentrations ≥ 100 nM (see Figure S2a).

### Ziram is Selectively Toxic to DA Neurons

**Figure 1:**
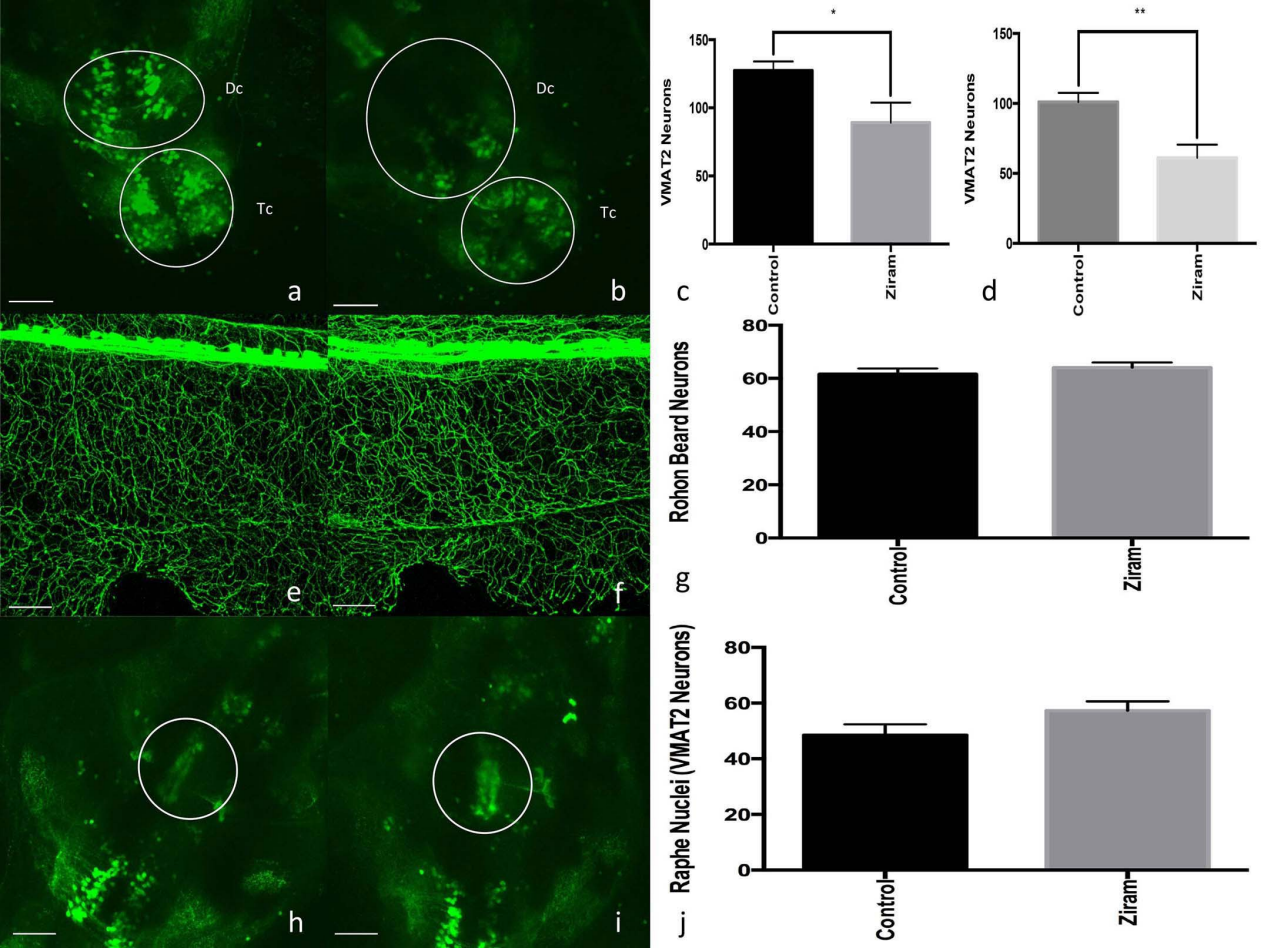
Ziram is selectively toxic to dopaminergic neurons. VMAT2 zebrafish (ZF) embryos were exposed to vehicle (*A*) and 50 nM ziram (*B*) at 24 hour post- fertilization (hpf). Neuronal counts for the telencephalic (TC) and diencephalic (DC) clusters were conducted at 5 days post-fertilization (dpf) (*n* = 5). A 30% decrease in telencephalic neurons (*C*) and a 39% decrease in diencephalic neurons (*D*) were observed in ziram-treated fish. Compared with controls (*E*), ziram’s toxicity did not extend to Rohon-Beard neurons (5 dpf, *n* = 7) after exposure to 50 nM ziram (*F,G*). Raphe nuclei neurons are also labeled in VMAT2:GFP fish (serotonergic neurons), and compared with controls (*H*), they were unaffected by ziram exposure (*I*). Raphe nuclei neuron counts are shown in (*J*). Scale bar = 500 μm. *, *p* < 0.05; **, *p* < 0.01. Two-tailed Student’s *t*-test.

We previously reported that ziram was selectively toxic to DA neurons in rat primary mesencephalic cultures (Chou et al. 2008). To determine if ziram is also toxic to DA neurons *in vivo*, we utilized a VMAT2:GFP ZF line that expresses GFP driven by the VMAT2 promoter to monitor aminergic neuronal integrity as previously described (Fitzmaurice et al. 2013; Wen et al. 2008). We tested ziram exposure at a concentration of 50 nM because this was the highest concentration at which there was no significant degree of lethality. A decrease in the number of GFP-labeled neurons was observed in the telencephalic (TC) and diencephalic (DC) clusters (Figure 1a,b,c,d) in embryos treated with ziram. These clusters are predominantly DA, although they also include some noradrenergic neurons (Pasterkamp et al. 2009; Rink and Wullimann 2002). The toxicity of ziram to DA neurons was further supported by the measured 63% decrease in tyrosine hydroxylase-1 (TH-1) protein levels (see Figure S3). To determine whether this toxicity was selective to aminergic neurons, we measured the integrity of Rohon-Beard neurons after exposure to 50 nM ziram using Tg(sensory:GFP) embryos as previously described (Fitzmaurice et al. 2013; Sagasti et al. 2005). No significant change in the number of labeled sensory neurons was observed compared with vehicle controls (Figure 1e,f,g). Furthermore, ziram appeared selectively toxic to DA neurons because serotonergic neurons in the raphe nuclei of the VMAT2 transgenic line were unaffected by ziram exposure (Figure 1h–j).

### Ziram Alters ZF Swimming Behavior Caused By DA Dysfunction

**Figure 2:**
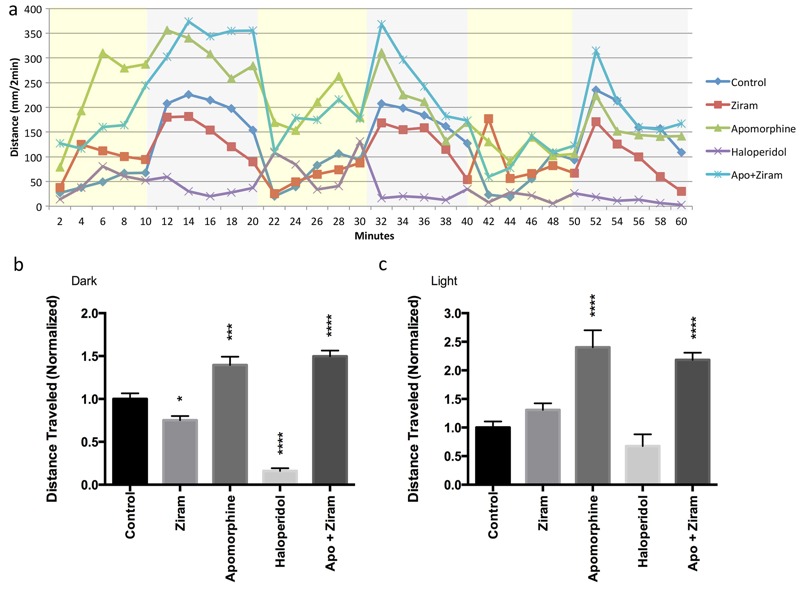
Ziram causes alterations in zebrafish (ZF) motor behavior. Movement was tracked for larval ZF at 7 days post-fertilization (dpf) (*n* = 24). Distances > 2 mm were tracked under an alternating light (yellow)/dark (gray) cycle (*A*). A 24.7% decrease (*p* < 0.05) in distance traveled during periods of dark was observed in ziram-treated ZF relative to vehicle-treated ZF (*B*), but no significant difference was observed during periods of light (*C*). A similar pattern of swimming, less in the dark but no difference in the light, was measured when ZF were treated with the dopamine antagonist haloperidol (*B,C*). The dopamine agonist apomorphine (Apo) increased swimming to a similar degree in the ziram-treated and control ZF, suggesting that the postsynaptic dopaminergic system remained intact. No significant difference in motor behavior was observed for ziram + apomorphine versus apomorphine alone for light or dark conditions. All treatment groups were compared with vehicle controls for statistical analysis. *, *p* < 0.05; ***, *p* < 0.001; ****p < 0.0001; one-way analysis of variance (ANOVA).

In light of the decrease in DA neuron number, we asked if there was a behavioral phenotype of ziram toxicity that could be attributed to DA neuronal loss. ZF embryos were treated with 50 nM ziram at 24 hpf, and swimming was measured at 7 dpf under alternating light and dark cycles (Figure 2a). Vehicle-treated ZF were much more active in the dark in a stereotypical manner, as previously described (Burgess and Granato 2007; Farrell et al. 2011). In the dark, ziram-treated fish swam significantly less than controls, but no change was seen in the light (Figure 2b,c). To determine whether these behavioral changes were due to DA neuronal dysfunction, we treated controls with the dopamine antagonist haloperidol and found a similar pattern to that observed with ziram treatment (i.e., less swimming distance in the dark and no difference in the light). The decrease in swimming in ziram-treated fish appeared to be secondary to a presynaptic dopamine loss because apomorphine, a potent agonist of the dopamine receptors, stimulated swimming equally in ziram-treated and vehicle-treated ZF (Figure 2b,c).

### ZF *γ* 1 Synuclein Forms Aggregates *in Vitro* and *in Vivo*

α-Syn aggregation appears to be central to the pathophysiology of PD. Ziram increases α-syn levels in DA neurons in primary mesencephalic cultures likely through inhibition of the proteasome (Chou et al. 2008; Wang et al. 2006). Three isoforms of synuclein (β, γ1, and γ2) have previously been partially characterized in ZF, but none appears to be the exact orthologue to human α-syn (Milanese et al. 2012; Sun and Gitler 2008). ZF γ1 has been previously demonstrated to be expressed widely throughout the developing ZF brain (Chen et al. 2009). Because ZF γ1 synuclein contains N-terminal repeats and hydrophobic regions similar to those of α-syn, and because expression of human α-syn rescues behavioral defects of γ1-deficient ZF embryos (Milanese et al. 2012), we investigated the propensity of ZF γ1 to aggregate and induce toxicity *in vitro* and *in vivo*.

**Figure 3:**
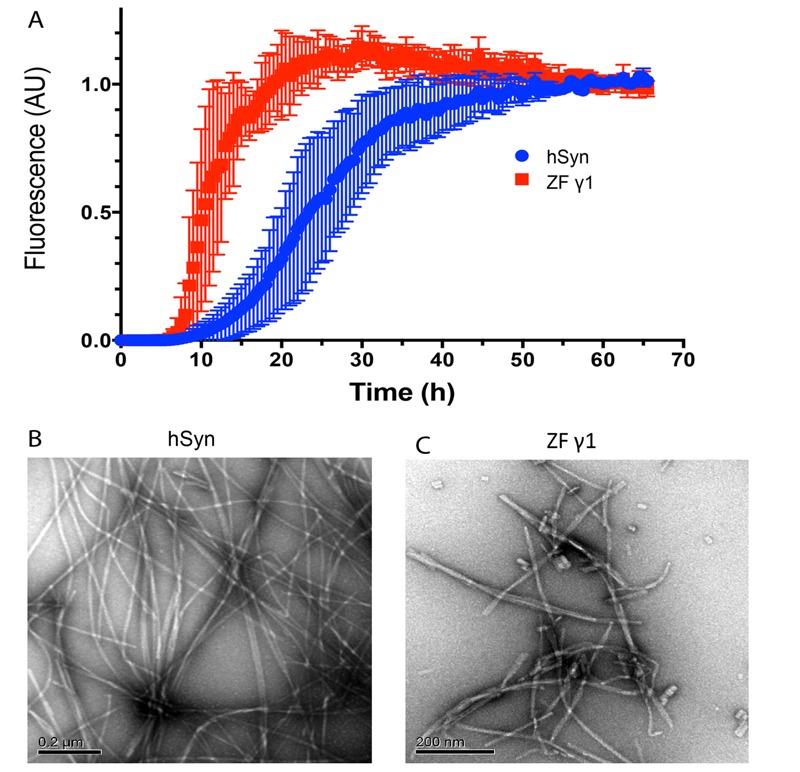
ZF γ1 synuclein aggregates and forms fibrils similarly to human α-synuclein (α*-*syn). Recombinant human α-syn (hSyn) or ZF γ1 (both 150 μM) were incubated over a 66-hr period, and thioflavin T fluorescence was monitored (*A*). The morphology of hSyn (*B*) and ZF γ1 (*C*) was examined using transmission electron microscopy (TEM) at the end of the aggregation reaction (scale bar = 0.2 μm).

Recombinant human α-syn and ZF γ1 were incubated and monitored for β-sheet formation using ThT fluorescence. The morphology of each sample was examined using TEM at the end of the aggregation reactions (Sinha et al. 2012). The ThT fluorescence in the α-syn samples began to increase following a lag phase of ~10 hr and reached a plateau at ~55 hr (Figure 3A). In the ZF γ1 samples, the lag phase was shorter, ~7 hr, and the kinetics of the ThT fluorescence increase were faster, reaching a plateau by ~25 hr (Figure 3A). Both proteins formed abundant fibrils as determined by TEM, although the fibrils differed in length. α-Syn formed long fibrils that were ≥ 1 μm in length with a diameter of 10 ± 1 nm (Figure 3B), whereas ZF γ1 formed 20- to 740-nm-long fibrils with a diameter of 9 ± 2 nm (Figure 3C). These differences in the length and abundance of the fibrils can be attributed to ZF γ1’s faster nucleation rate, as reflected by the shorter ThT fluorescence lag phase relative to that of α-syn. Thus, similarly to α-syn and other amyloidogenic proteins, ZF γ1 is an amyloidogenic protein that has the ability to form β-sheet-rich fibrils.

**Figure 4:**
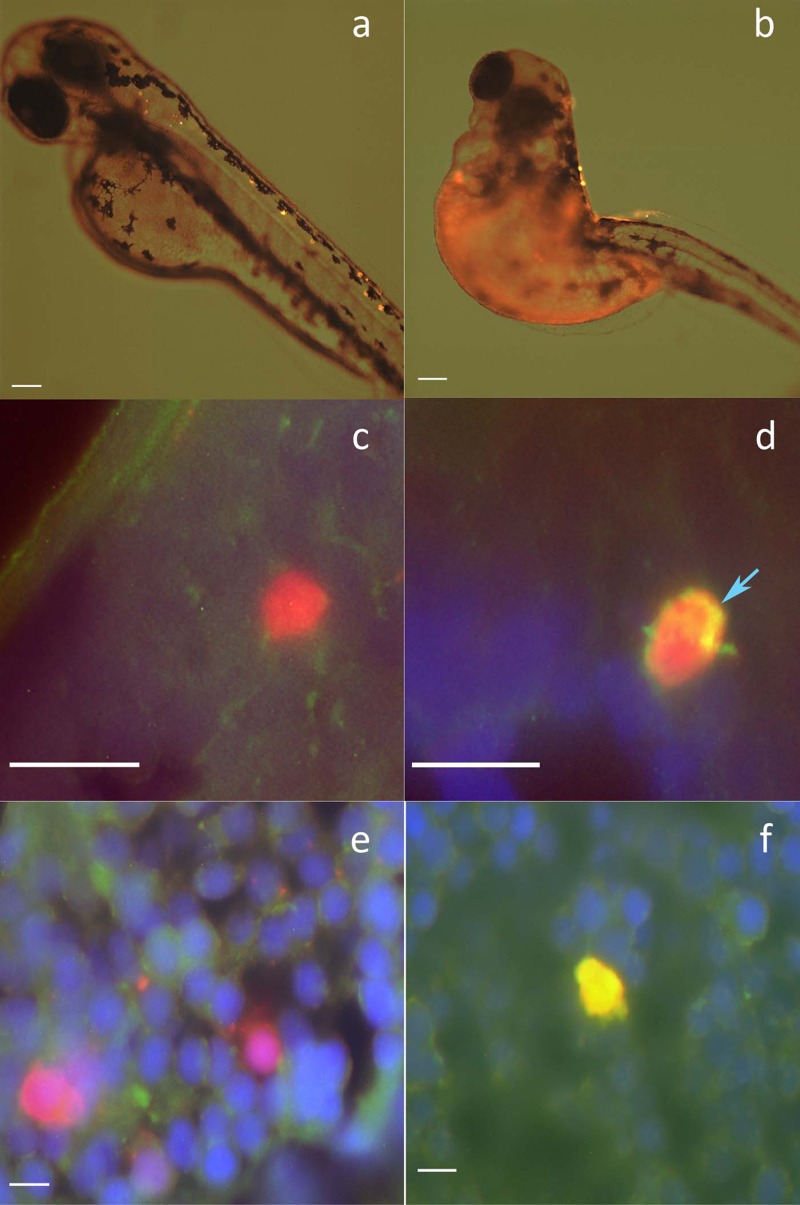
Overexpression of ZF γ1 *in vivo* causes intracellular aggregation of synuclein. Embryos were injected with constructs for *HuC*-T2A-DsRed (*A*) or *HuC*-γ1-T2A-DsRed (*B*); embryos shown at 2 days post-fertilization (dpf) (scale bar = 100 μm). Sectioned embryos were stained with a primary antibody for ZF γ1. *HuC*-DsRed–injected (*C*) embryos did not have intracellular ZF γ1 aggregates, whereas embryos injected with *HuC*-ZFγ1 (*D*) were found to have intracellular ZF γ1 aggregates (blue arrow) (scale bar = 10 μm). To determine if ZF γ1 formed β-pleated sheets *in vivo*, embryos injected with *HuC*-DsRed and *HuC*-γ1-T2A-DsRed were stained with thioflavin S (ThS) (scale bar = 10 μm). Only neurons overexpressing ZF γ1 (*f*) were ThS-positive (*E*).

We previously demonstrated that over-expression of α-syn in ZF neurons led to malformed embryos, intracellular aggregates, and neuronal death (Prabhudesai et al. 2012). Here, we over-expressed ZF γ1 in ZF neurons to determine whether increased levels of endogenous protein induced aggregate formation and were neurotoxic *in vivo*. In this model, ZF γ1 was expressed under the control of the *HuC* neuronal promoter as a fusion protein with T2A-DsRed. The T2A peptide is posttranslationally cleaved, releasing native ZF γ1 and the fluorescent DsRed reporter in equal molar concentrations (Prabhudesai et al. 2012). Embryos over-expressing ZF γ1 were malformed and showed reduced survival compared with embryos that over-expressed DsRed (Figure 4a,b). Using an antibody specific for ZF γ1, we found that embryos over-expressing ZF γ1 formed intracytoplasmic aggregates (Figure 4c,d). Additionally, ThS staining was observed only in neurons over-expressing ZF γ1, whereas no staining was seen in DsRed-expressing controls (Figure 4e,f) indicating that these aggregates contained β-sheet-rich aggregates.

**Figure 5:**
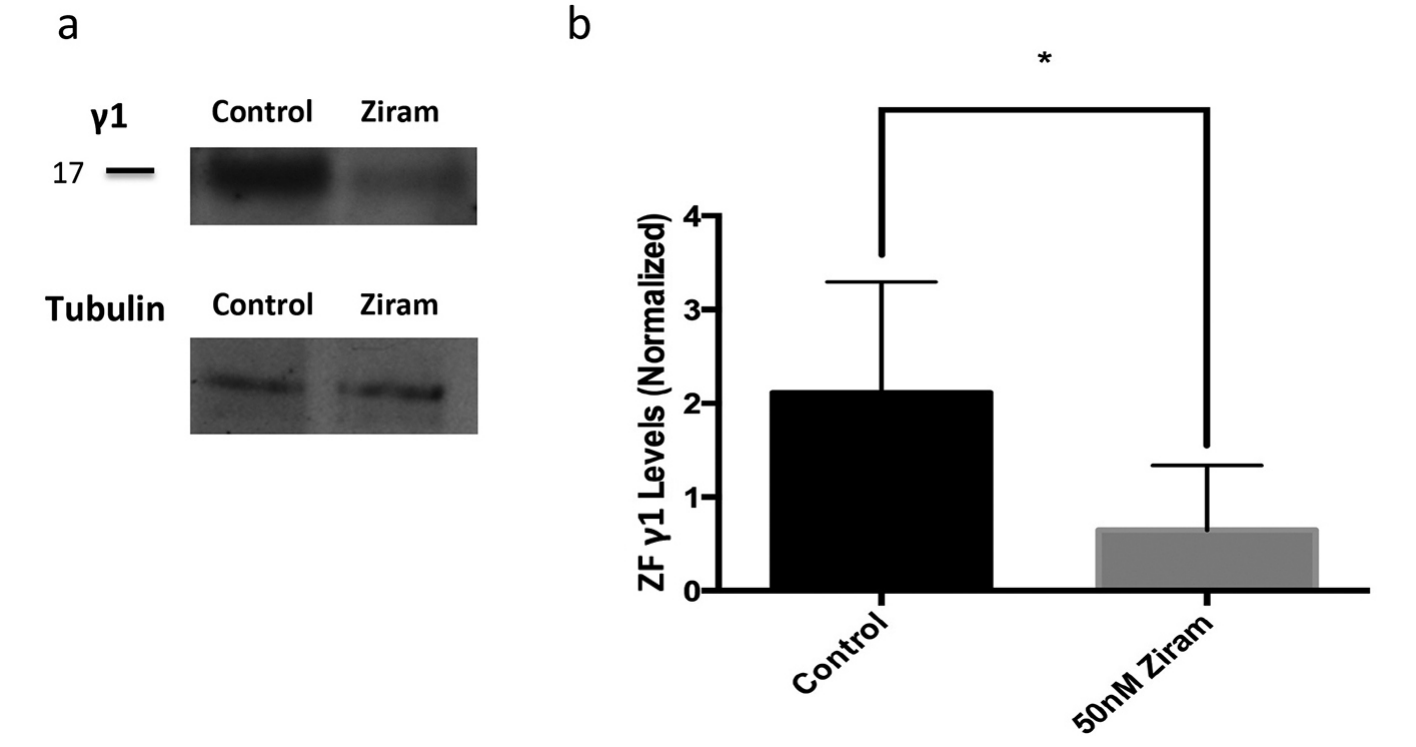
Ziram decreases ZF γ1 levels. Embryos were treated with ziram or vehicle, and ZF γ1 levels were determined by Western blot analysis at 5 days post-fertilization (dpf) (normalized to tubulin). A 69.3% decrease in band density for ZF γ1 was observed for samples treated with 50 nM ziram (*A*,*B*, *n* = 4). *, *p* < .05. Two-tailed Student’s *t*-test.

Because ziram increases α-syn in primary neuronal cultures, and because ZF γ1 displays many of the same characteristics as α-syn, we determined the effects of ziram on ZF γ1 levels at 5 dpf. Western blot analysis of ZF embryo extracts probed with anti-ZF γ1 antibody revealed a band at 17 kDa (presumed monomer, Figure 5a; see also Figure S1a). A significant decrease in the intensity of the 17 kDa band was observed for embryos treated with 50 nM ziram compared with vehicle-treated ZF (Figure 5b). Western blots conducted under native conditions revealed a major band at 480 kDa and a minor band at 242 kDa (see Figure S1b) for both ziram-treated and control samples, suggesting that ziram does not alter the degree of oligomerization of ZF γ1.

### Ziram’s Toxicity is ZF *γ* 1-Dependent

**Figure 6:**
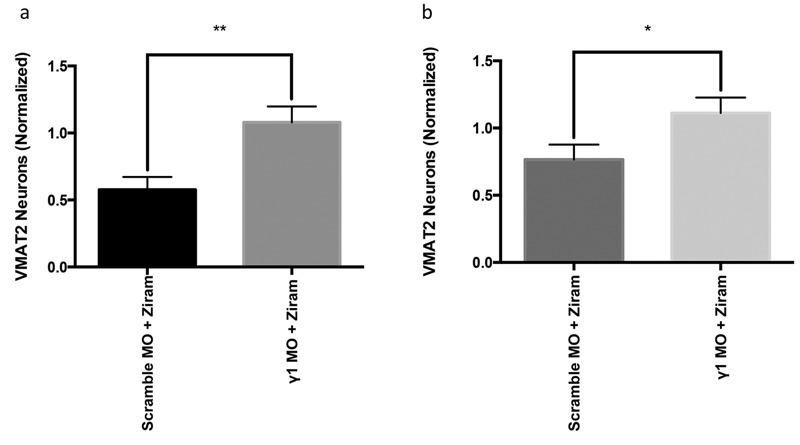
Ziram’s toxicity is ZF γ1-dependent. ZF γ1 expression was reduced using a specific morpholino (MO), and a scrambled MO was used as a control. Ziram treatment began at 24 hour post-fertilization (hpf). Neuronal counts (normalized to vehicle + scramble/γ1 MO) for γ1 MO-injected embryos (*n* = 14). An 86.8% increase in labeled telencephalic VMAT2 neurons (*A*) and a 45.1% increase in labeled diencepahlic VMAT2 neurons (*B*) at 3 dpf was observed for fish treated with ziram + γ1 MO versus ziram + scramble MO. *, *p* < 0.05; **, *p* < 0.01. Student’s *t*-test.

To understand the role of ZF γ1 in ziram’s neuronal toxicity, we knocked down ZF γ1 expression with MOs before ziram treatment. MO knockdown of ZF γ1 protected telencephalic and diencephalic aminergic neurons from ziram’s toxicity (Figure 6a,b).

**Figure 7:**
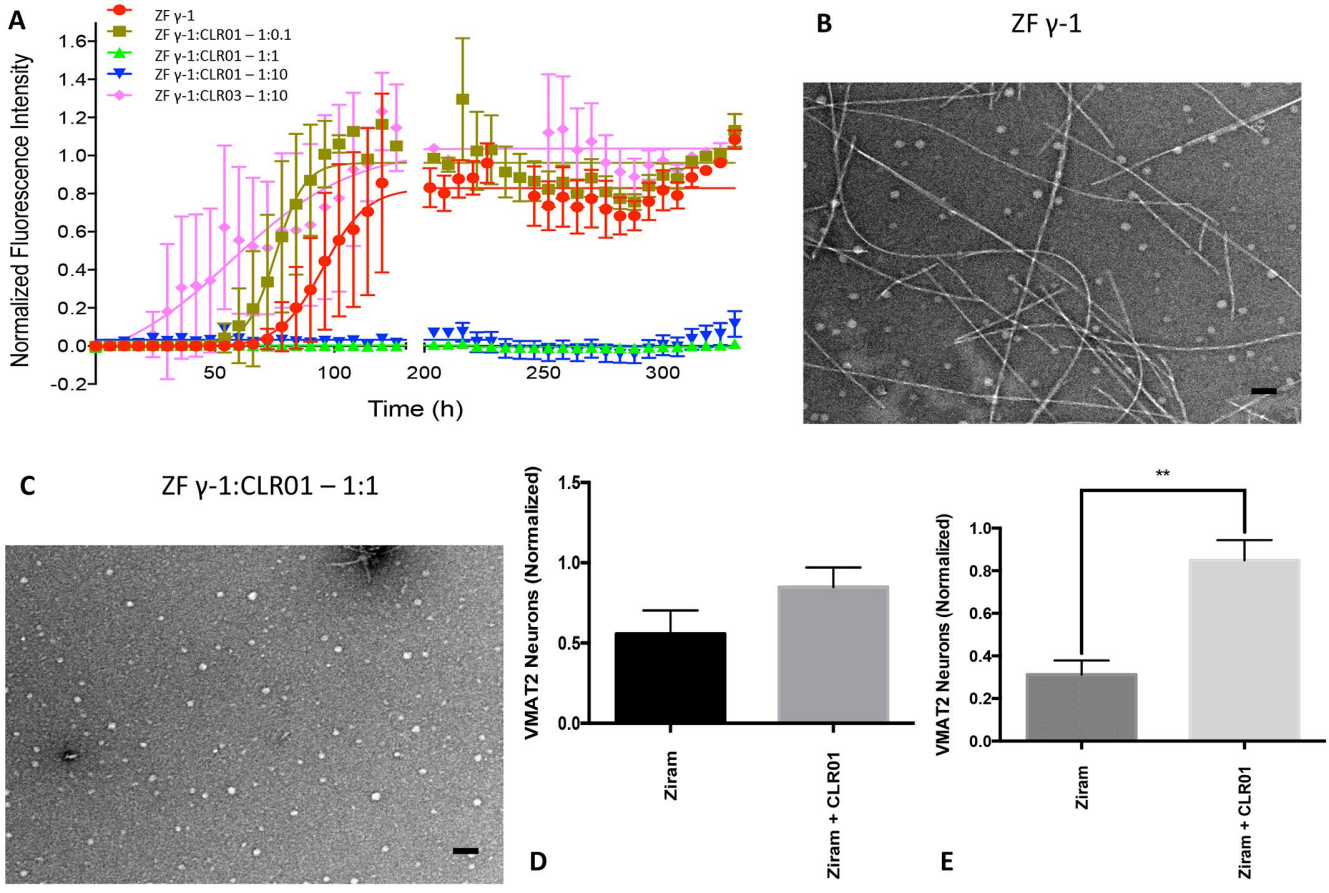
CLR01 inhibits ZF γ1 aggregation *in vitro* and protects against ziram’s toxicity *in vivo*. Time-dependent change in normalized thioflavin T (ThT) fluorescence over 14 days for 100 μM γ1 incubated in the absence or presence of CLR01 (*A*). The first and last 130 hr are shown (*n *= 4). Electron micrographs of ZF γ1 alone (*B*) and ZF γ1 with CLR01 (*C*) in an equimolar ratio (scale bar = 100 nm) obtained on day 9 or day 10 of each reaction [analysis of variance (ANOVA), *p* < 0.0001]. CLR01 treatment protected against ziram toxicity (50 nM; *n *= 6) to VMAT2 neurons in the diencephalic cluster (*E*), but the difference did not reach statistical significance in the telencephalic cluster (*D*) at 5 days post-fertilization (dpf). **, *p* < 0.01; Student’s *t*-test.

To further explore the role of ZF γ1 in ziram’s neuronal toxicity, we utilized the molecular tweezer CLR01. CLR01 has previously been found to reduce human α-syn toxicity by disrupting α-syn aggregation *in vivo* and *in vitro* (Acharya et al. 2014; Prabhudesai et al. 2012; Sinha et al. 2012). We have previously reported that CLR01 reduced α-syn toxicity in cell-based models and in ZF (Prabhudesai et al. 2012). Here, we found that CLR01 also inhibited the formation of recombinant ZF γ1 fibrils, as determined by ThT fluorescence and EM (Figure 7A–C), and it reduced ziram neurotoxicity *in vivo* as measured by the integrity of VMAT2:GFP neurons (Figure 7D). Thus, ziram’s neurotoxicity appears to be dependent not only on total ZF γ1 levels, as suggested by the MO experiment (Figure 6), but also on the formation of toxic forms of ZF γ1, which was disrupted by CLR01 (Figure 7).

## Discussion

The link between pesticide exposure and PD has been increasingly recognized in recent years. The identification of specific toxins that increase PD risk such as paraquat, rotenone, ziram, and maneb (Gatto et al. 2009; Tanner et al. 2009, 2011; Wang et al. 2011), has facilitated mechanistic studies. Ziram has been shown to inhibit both the UPS and ALDH (Chou et al. 2008; Fitzmaurice et al. 2013, 2014), but a direct link between these activities and neuronal toxicity *in vivo* has not yet been established. In this study, we used a ZF model to study environmental toxins relevant to PD and found that ziram caused selective DA neuron damage with behavioral consequences. Because human α-syn is central to the pathogenesis of PD, we hypothesized that ZF γ1 mediates ziram’s neurotoxicity. In support of this hypothesis, we found that ZF γ1 forms fibrils and is neurotoxic in a manner similar to that of human α-syn and that ziram’s toxicity was ZF γ1-dependent.

Although ZF offer several advantages over other animal models when studying environmental toxins, there are a number of limitations that need to be considered. In all of our studies, we used developing embryos, whereas PD is a disease of aging. Embryos were exposed at 24 hpf, and formation of the blood–brain barrier begins at approximately 3 dpf (Fleming et al. 2013). Additionally, the metabolism of ziram might be different in ZF embryos than in mammals because absorption at this early age may occur orally, dermally, or through the yolk. Despite these limitations, we believe our model is valid because the results are consistent with those of previous studies performed in rodents where ziram and maneb have been shown to cause selective DA cell loss (Chou et al. 2008; Thiruchelvam et al. 2000).

The behavioral effect of ziram on ZF embryos appears to be caused by a specific deficiency in DA signaling and not simply by nonspecific toxicity. Ziram decreased locomotion in the dark but not in the light in a similar manner to that of the dopamine antagonist haloperidol. This decrease in activity was reversed by the dopamine agonist apomorphine, suggesting that the behavioral phenotype was caused by a presynaptic dopamine deficiency. These results are consistent with the apparent loss of VMAT2:GFP neurons. Less-specific neurotoxins decrease locomotion in both the light and the dark and would not be expected to be completely reversed by a dopamine agonist. The selective loss of DA neurons caused by ziram is therefore supported by both the behavioral data and the preservation of non-DA neurons (Rohon-Beard and serotonergic neurons of the raphe nuclei). It is possible that the loss of the GFP signal does not reflect neuron loss but simply injury, yet the behavioral changes and the decrease in TH-1 support the hypothesis that ziram caused DA neuron injury and dysfunction.

It is not completely clear how ziram causes selective DA neuron damage, but the mechanism appears to require ZF γ1. Others have previously shown that ZF γ1 might have a similar physiologic function to that of human α-syn (Milanese et al. 2012), but we were surprised to find that ZF γ1 is neurotoxic like α-syn. Here, we demonstrated that recombinant ZF γ1 formed amyloid fibrils with kinetics similar to those of α-syn, and over-expression of ZF γ1 in neurons led to the formation of aggregates and neurotoxicity in a manner almost identical to that of human α-syn (Prabhudesai et al. 2012).

Previously, treatment with ziram was shown to inhibit the proteasome and to increase accumulation of α-syn in primary neuronal cultures (Chou et al. 2008; Wang et al. 2006). Interestingly, we observed a decrease in protein levels of ZF γ1 in ziram-treated embryos. The differences between these results are likely a result of the different models used, the different exposure times, and the different methods used to measure synuclein levels. In the neuronal culture study, ziram exposures were much shorter (48 hr) than those in the present study, and synuclein levels were determined only in the soma of tyrosine hydroxylase–positive neurons. Because ziram decreases protein degradation by inhibiting the proteasome, we hypothesized that synuclein accumulated in the surviving neurons. In the present study, we measured total embryonic synuclein by Western blot analysis. Because synuclein is primarily a synaptic protein in differentiated neurons *in vivo*, we hypothesize that the lower levels we measured after 120 hr of treatment were due to neuronal terminal loss. In the present study, DA terminal loss was confirmed by the reduction of GFP fluorescence in VMAT-2 neurons and the reduction of TH immunoreactivity, and by behavioral abnormalities that were reversed by a dopamine agonist. The fact that reducing ZF γ1 levels with MOs almost completely protected against ziram toxicity suggests that ziram increased the formation of toxic ZF γ1 species early in embryonic development. Over-expression of ZF γ1 in neurons did lead to the formation of β-sheet-rich aggregates, but we did not detect fibril formation with ThS *in vivo* after ziram exposure. The formation of synuclein β-sheets is dependent on several factors, including concentration and time. In ziram-treated embryos, ZF γ1 might not have reached sufficiently high concentrations or had enough time to form aggregates. Regardless, others have suggested that prefibrillar oligomers of α-synuclein may be the cause of DA neuronal toxicity (Danzer et al. 2007; Winner et al. 2011). These data suggest that the toxic effect of ziram might be mediated by toxic, soluble ZF γ1 oligomers. This hypothesis is supported by the finding that CLR01 also protected against ziram toxicity. CLR01 has previously been shown to act on amyloidogenic proteins and to protect against their toxicity both *in vivo* and *in vitro* (Attar et al. 2012; Prabhudesai et al. 2012; Sinha et al. 2011). The only known activities of CLR01 are remodeling the self-assembly of amyloidogenic proteins into nontoxic and nonamyloidogenic structures and facilitating their clearance (Acharya et al. 2014; Attar and Bitan 2014; Prabhudesai et al. 2012).

Most neurodegenerative disorders like PD have a relatively selective pattern of neuronal loss, although the mechanisms responsible for this selectivity have remained elusive. Ziram and other DTCs have been shown to inhibit the UPS, but this would not explain the selectivity of the neuronal damage. It is possible that ziram’s ability to inhibit ALDH in addition to its UPS-inhibiting activity lead to selective DA damage. ALDH inhibition causes the accumulation of 3,4-dihydroxy​phenylacetaldehyde (DOPAL) in DA neurons, which is toxic (Burke et al. 2008; Casida et al. 2014; Fitzmaurice et al. 2013, 2014; Panneton et al. 2010). The ZF model described here offers a powerful tool for further mechanistic studies.

## Conclusions

In summary, we report that ziram, a pesticide that increases the risk of PD, causes selective DA neuron damage that is synuclein-dependent in ZF. These findings provide potentially important mechanistic implications on how ziram and possibly other environmental toxins can contribute to the pathogenesis of neurodegenerative disorders such as PD. A better understanding of these processes has the potential to illuminate critical pathways in disease formation and to highlight critical targets for future therapeutic exploitation.

## Supplemental Material

(2.3 MB) PDF

## References

[r1] Acharya S, Safaie BM, Wongkongkathep P, Ivanova MI, Attar A, Klärner FG (2014). Molecular basis for preventing alpha-synuclein aggregation by a molecular tweezer.. J Biol Chem.

[r2] Attar A, Bitan G (2014). Disrupting self-assembly and toxicity of amyloidogenic protein oligomers by “molecular tweezers” - from the test tube to animal models.. Curr Pharm Des.

[r3] Attar A, Ripoli C, Riccardi E, Maiti P, Li Puma DD, Liu T (2012). Protection of primary neurons and mouse brain from Alzheimer’s pathology by molecular tweezers.. Brain.

[r4] Baba M, Nakajo S, Tu PH, Tomita T, Nakaya K, Lee VM (1998). Aggregation of alpha-synuclein in Lewy bodies of sporadic Parkinson’s disease and dementia with Lewy bodies.. Am J Pathol.

[r5] Barnhill LM, Bronstein JM (2014). Pesticides and Parkinson’s disease: is it in your genes?. Neurodegener Dis Manag.

[r6] Betarbet R, Sherer TB, MacKenzie G, Garcia-Osuna M, Panov AV, Greenamyre JT (2000). Chronic systemic pesticide exposure reproduces features of Parkinson’s disease.. Nat Neurosci.

[r7] Biancalana M, Koide S (2010). Molecular mechanism of Thioflavin-T binding to amyloid fibrils.. Biochim Biophys Acta.

[r8] Bretaud S, Li Q, Lockwood BL, Kobayashi K, Lin E, Guo S (2007). A choice behavior for morphine reveals experience-dependent drug preference and underlying neural substrates in developing larval zebrafish.. Neuroscience.

[r9] Briquet M, Sabadie-Pialoux N, Goffeau A (1976). Ziram, a sulfhydryl reagent and specific inhibitor of yeast mitochondrial dehydrogenases.. Arch Biochem Biophys.

[r10] Brown TP Rumsby PC Capleton AC Rushton L Levy LS 2006 Pesticides and Parkinson’s disease—is there a link? Environ Health Perspect 114 156 164, doi:10.1289/ehp.8095 16451848 PMC1367825

[r11] Burgess HA, Granato M (2007). Modulation of locomotor activity in larval zebrafish during light adaptation.. J Exp Biol.

[r12] Burke WJ, Kumar VB, Pandey N, Panneton WM, Gan Q, Franko MW (2008). Aggregation of α-synuclein by DOPAL, the monoamine oxidase metabolite of dopamine.. Acta Neuropathol.

[r13] Casida JE, Ford B, Jinsmaa Y, Sullivan P, Cooney A, Goldstein DS (2014). Benomyl, aldehyde dehydrogenase, DOPAL, and the catecholaldehyde hypothesis for the pathogenesis of Parkinson’s disease.. Chem Res Toxicol.

[r14] Chartier-Harlin MC, Kachergus J, Roumier C, Mouroux V, Douay X, Lincoln S (2004). α-Synuclein locus duplication as a cause of familial Parkinson’s disease.. Lancet.

[r15] Chen YC, Cheng CH, Chen GD, Hung CC, Yang CH, Hwang SP (2009). Recapitulation of zebrafish *sncga* expression pattern and labeling the habenular complex in transgenic zebrafish using green fluorescent protein reporter gene.. Dev Dyn.

[r16] Chou AP, Maidment N, Klintenberg R, Casida JE, Li S, Fitzmaurice AG (2008). Ziram causes dopaminergic cell damage by inhibiting E1 ligase of the proteasome.. J Biol Chem.

[r17] Danzer KM, Haasen D, Karow AR, Moussaud S, Habeck M, Giese A (2007). Different species of α-synuclein oligomers induce calcium influx and seeding.. J Neurosci.

[r18] Dorsey ER, Constantinescu R, Thompson JP, Biglan KM, Holloway RG, Kieburtz K (2007). Projected number of people with Parkinson disease in the most populous nations, 2005 through 2030.. Neurology.

[r19] Farrell TC, Cario CL, Milanese C, Vogt A, Jeong JH, Burton EA (2011). Evaluation of spontaneous propulsive movement as a screening tool to detect rescue of Parkinsonism phenotypes in zebrafish models.. Neurobiol Dis.

[r20] Farrer M, Kachergus J, Forno L, Lincoln S, Wang DS, Hulihan M (2004). Comparison of kindreds with parkinsonism and α-synuclein genomic multiplications.. Ann Neurol.

[r21] Feany MB, Bender WW (2000). A *Drosophila* model of Parkinson’s disease.. Nature.

[r22] Fitzmaurice AG, Rhodes SL, Cockburn M, Ritz B, Bronstein JM (2014). Aldehyde dehydrogenase variation enhances effect of pesticides associated with Parkinson disease.. Neurology.

[r23] Fitzmaurice AG, Rhodes SL, Lulla A, Murphy NP, Lam HA, O’Donnell KC (2013). Aldehyde dehydrogenase inhibition as a pathogenic mechanism in Parkinson disease.. Proc Natl Acad Sci U S A.

[r24] Fleming A Diekmann H Goldsmith P 2013 Functional characterisation of the maturation of the blood-brain barrier in larval zebrafish. PLoS One 8 e77548, doi:10.1371/journal.pone.0077548 24147021 PMC3797749

[r25] Fokkens M, Schrader T, Klärner FG (2005). A molecular tweezer for lysine and arginine.. J Am Chem Soc.

[r26] Gatto NM Cockburn M Bronstein J Manthripragada AD Ritz B 2009 Well-water consumption and Parkinson’s disease in rural California. Environ Health Perspect 117 1912 1918, doi:10.1289/ehp.0900852 20049211 PMC2799466

[r27] Gatto NM, Rhodes SL, Manthripragada AD, Bronstein J, Cockburn M, Farrer M (2010). α-Synuclein gene may interact with environmental factors in increasing risk of Parkinson’s disease.. Neuroepidemiology.

[r28] Goldman SM (2014). Environmental toxins and Parkinson’s disease.. Annu Rev Pharmacol Toxicol.

[r29] Goldman SM, Kamel F, Ross GW, Jewell SA, Bhudhikanok GS, Umbach D (2012). Head injury, alpha-synuclein Rep1, and Parkinson’s disease.. Ann Neurol.

[r30] Gunnarsson L, Jauhiainen A, Kristiansson E, Nerman O, Larsson DG (2008). Evolutionary conservation of human drug targets in organisms used for environmental risk assessments.. Environ Sci Technol.

[r31] Haendel MA, Tilton F, Bailey GS, Tanguay RL (2004). Developmental toxicity of the dithiocarbamate pesticide sodium metam in zebrafish.. Toxicol Sci.

[r32] Hope AD, Myhre R, Kachergus J, Lincoln S, Bisceglio G, Hulihan M (2004). α-Synuclein missense and multiplication mutations in autosomal dominant Parkinson’s disease.. Neurosci Lett.

[r33] Ibáñez P, Bonnet AM, Débarges B, Lohmann E, Tison F, Pollak P (2004). Causal relation between α-synuclein gene duplication and familial Parkinson’s disease.. Lancet.

[r34] International Parkinson Disease Genomics Consortium, Nalls MA, Plagnol V, Hernandez DG, Sharma M, Sheerin UM, et al (2011). Imputation of sequence variants for identification of genetic risks for Parkinson’s disease: a meta-analysis of genome-wide association studies.. Lancet.

[r35] Joyner SB (2015). Letter from SB Joyner, Fungicide-Herbicide Branch, Registration Divison 7505P, Office of Chemical Safety and Pollution Prevention, U.S. Environmental Protection Agency, to SB Hutcheson, Regulatory Affairs Manager, United Phosphorus, Inc. Label amendment – remove use on blackberry on the label per EPA request. Product name: Ziram 76Df Fungicide. EPA Registration Number: 70506-173, 2 October 2015.. http://www3.epa.gov/pesticides/chem_search/ppls/070506-00173-20151002.pdf.

[r36] Krüger R, Kuhn W, Müller T, Woitalla D, Graeber M, Kösel S (1998). Ala30Pro mutation in the gene encoding α-synuclein in Parkinson’s disease.. Nat Genet.

[r37] Maraganore DM, de Andrade M, Elbaz A, Farrer MJ, Ioannidis JP, Krüger R (2006). Collaborative analysis of α-synuclein gene promoter variability and Parkinson disease.. JAMA.

[r38] McCormack AL, Thiruchelvam M, Manning-Bog AB, Thiffault C, Langston JW, Cory-Slechta DA (2002). Environmental risk factors and Parkinson’s disease: selective degeneration of nigral dopaminergic neurons caused by the herbicide paraquat.. Neurobiol Dis.

[r39] Milanese C, Sager JJ, Bai Q, Farrell TC, Cannon JR, Greenamyre JT (2012). Hypokinesia and reduced dopamine levels in zebrafish lacking β- and γ1-synucleins.. J Biol Chem.

[r40] Nishioka K, Hayashi S, Farrer MJ, Singleton AB, Yoshino H, Imai H (2006). Clinical heterogeneity of α-synuclein gene duplication in Parkinson’s disease.. Ann Neurol.

[r41] Panneton WM Kumar VB Gan Q Burke WJ Galvin JE 2010 The neurotoxicity of DOPAL: behavioral and stereological evidence for its role in Parkinson disease pathogenesis. PLoS One 5 e15251, doi:10.1371/journal.pone.0015251 21179455 PMC3001493

[r42] Pasterkamp RJ, Smidt MP, Burbach JPH (2009). *Development and Engineering of Dopamine Neurons*..

[r43] Polymeropoulos MH, Lavedan C, Leroy E, Ide SE, Dehejia A, Dutra A (1997). Mutation in the α-synuclein gene identified in families with Parkinson’s disease.. Science.

[r44] Prabhudesai S, Sinha S, Attar A, Kotagiri A, Fitzmaurice AG, Lakshmanan R (2012). A novel “molecular tweezer” inhibitor of α-synuclein neurotoxicity *in vitro* and *in vivo*.. Neurotherapeutics.

[r45] Rasband WS (1997–2016). ImageJ.. http://imagej.nih.gov/ij/.

[r46] Rink E, Wullimann MF (2002). Development of the catecholaminergic system in the early zebrafish brain: an immunohistochemical study.. Brain Res Dev Brain Res.

[r47] Ritz B Rhodes SL Bordelon Y Bronstein J 2012 α-Synuclein genetic variants predict faster motor symptom progression in idiopathic Parkinson disease. PLoS One 7 e36199, doi:10.1371/journal.pone.0036199 22615757 PMC3352914

[r48] Ryan SD, Dolatabadi N, Chan SF, Zhang X, Akhtar MW, Parker J (2013). Isogenic human iPSC Parkinson’s model shows nitrosative stress-induced dysfunction in MEF2-PGC1α transcription.. Cell.

[r49] Sagasti A, Guido MR, Raible DW, Schier AF (2005). Repulsive interactions shape the morphologies and functional arrangement of zebrafish peripheral sensory arbors.. Curr Biol.

[r50] Schweitzer J, Lohr H, Filippi A, Driever W (2012). Dopaminergic and noradrenergic circuit development in zebrafish.. Dev Neurobiol.

[r51] Sheng D Qu D Kwok KH Ng SS Lim AY Aw SS 2010 Deletion of the WD40 domain of LRRK2 in zebrafish causes Parkinsonism-like loss of neurons and locomotive defect. PLoS Genet 6 e1000914, doi:10.1371/journal.pgen.1000914 20421934 PMC2858694

[r52] Singleton AB Farrer M Johnson J Singleton A Hague S Kachergus J 2003 α-Synuclein locus triplication causes Parkinson’s disease. Science 302 841, doi:10.1126/science.1090278 14593171

[r53] Sinha S, Du Z, Maiti P, Klärner FG, Schrader T, Wang C (2012). Comparison of three amyloid assembly inhibitors: the sugar *scyllo*-inositol, the polyphenol epigallocatechin gallate, and the molecular tweezer CLR01.. ACS Chem Neurosci.

[r54] Sinha S, Lopes DH, Du Z, Pang ES, Shanmugam A, Lomakin A (2011). Lysine-specific molecular tweezers are broad-spectrum inhibitors of assembly and toxicity of amyloid proteins.. J Am Chem Soc.

[r55] Spillantini MG, Schmidt ML, Lee VM, Trojanowski JQ, Jakes R, Goedert M (1997). α-Synuclein in Lewy bodies.. Nature.

[r56] Sun Z, Gitler AD (2008). Discovery and characterization of three novel synuclein genes in zebrafish.. Dev Dyn.

[r57] Talbiersky P, Bastkowski F, Klärner FG, Schrader T (2008). Molecular clip and tweezer introduce new mechanisms of enzyme inhibition.. J Am Chem Soc.

[r58] Tang W, Ehrlich I, Wolff SB, Michalski AM, Wölfl S, Hasan MT (2009). Faithful expression of multiple proteins via 2A-peptide self-processing: a versatile and reliable method for manipulating brain circuits.. J Neurosci.

[r59] Tanner CM Kamel F Ross GW Hoppin JA Goldman SM Korell M 2011 Rotenone, paraquat, and Parkinson’s disease. Environ Health Perspect 119 866 872, doi:10.1289/ehp.1002839 21269927 PMC3114824

[r60] Tanner CM, Ross GW, Jewell SA, Hauser RA, Jankovic J, Factor SA (2009). Occupation and risk of Parkinsonism: a multicenter case-control study.. Arch Neurol.

[r61] Teraoka H, Urakawa S, Nanba S, Nagai Y, Dong W, Imagawa T (2006). Muscular contractions in the zebrafish embryo are necessary to reveal thiuram-induced notochord distortions.. Toxicol Appl Pharmacol.

[r62] Thiruchelvam M, Richfield EK, Baggs RB, Tank AW, Cory-Slechta DA (2000). The nigrostriatal dopaminergic system as a preferential target of repeated exposures to combined paraquat and maneb: implications for Parkinson’s disease.. J Neurosci.

[r63] Trinh J, Farrer M (2013). Advances in the genetics of Parkinson disease.. Nat Rev Neurol.

[r64] Wang A, Costello S, Cockburn M, Zhang X, Bronstein J, Ritz B (2011). Parkinson’s disease risk from ambient exposure to pesticides.. Eur J Epidemiol.

[r65] Wang XF, Li S, Chou AP, Bronstein JM (2006). Inhibitory effects of pesticides on proteasome activity: implication in Parkinson’s disease.. Neurobiol Dis.

[r66] Wen L, Wei W, Gu W, Huang P, Ren X, Zhang Z (2008). Visualization of monoaminergic neurons and neurotoxicity of MPTP in live transgenic zebrafish.. Dev Biol.

[r67] Winner B, Jappelli R, Maji SK, Desplats PA, Boyer L, Aigner S (2011). In vivo demonstration that α-synuclein oligomers are toxic.. Proc Natl Acad Sci U S A.

[r68] Xi Y, Noble S, Ekker M (2011). Modeling neurodegeneration in zebrafish.. Curr Neurol Neurosci Rep.

