# Supplementary material for: Neurotoxicity of the Parkinson Disease-Associated Pesticide Ziram Is Synuclein-Dependent in Zebrafish Embryos
Source: Environ Health Perspect. 2016 Jun 15;124(11):1766–75. doi: 10.1289/EHP141 (PMC5089875; doi:10.1289/EHP141)
Supplement: (2.3 MB) PDF [file EHP141.s001.acco.pdf]

Environ Health Perspect
DOI: 10.1289/EHP141

Note to readers with disabilities: *EHP* strives to ensure that all journal content is accessible to all readers. However, some figures and Supplemental Material published in *EHP* articles may not conform to 508 standards due to the complexity of the information being presented. If you need assistance accessing journal content, please contact ehp508@niehs.nih.gov. Our staff will work with you to assess and meet your accessibility needs within 3 working days.

Supplemental Material

Neurotoxicity of the Parkinson's Disease-Associated Pesticide Ziram Is Synuclein-Dependent in Zebrafish Embryos

Aaron Lulla, Lisa Barnhill, Gal Bitan, Magdalena I. Ivanova, Binh Nguyen, Kelley O'Donnell , Mark C. Stahl, Chase Yamashiro, Frank-Gerrit Klärner, Thomas Schrader, Alvaro Sagasti, and Jeff M. Bronstein

Table of Contents

Figure S1: ZF γ1 antibody is specific for ZF. Using denaturing conditions/SDS PAGE, a band for γ1 was detected at 17 kDa for ZF adult brain (ZF) and purified ZF γ1 (a). No ZF γ1 signal was detected for mouse brain (MB). Using non-denaturing conditions/Native PAGE, a major band for ZF γ1 was detected at 480 kDa and a minor band at 242 kDa (b). Peptide preincubated with the ZF γ1 antibody is shown for SDS PAGE. No band for ZF γ1 was detected after preincubation of γ1 antibody with γ1 peptide (c). ZF γ1 MO reduced protein levels of ZF γ1 as compared to scramble MO as determined by SDS PAGE (d).

Figure S2: Ziram is toxic in a concentration-dependent manner and causes notochord distortion. A significant change in toxicity was observed for embryos treated (24hpf) with 100nM and 1μM ziram by day 7 (n = 50, p<.0001) (a). ZF embryos treated with 50 nM ziram at 5 hpf, were found to have a shorter body axis, pericardial edema, and notochord distortion (b) as compared to vehicle treated ZF (c). p<.0001 Log-Rank test.

Figure S3: Ziram treatment results in reduction of TH-1 levels. Using Western blot analysis, the effect of ziram (50nM) on TH-1 levels was investigated. A 63% reduction in TH-1 was observed for embryos treated with 50nM ziram vs. controls (n=4; *p-value = .03 using two-tailed T test). Bars represent standard error of the mean.

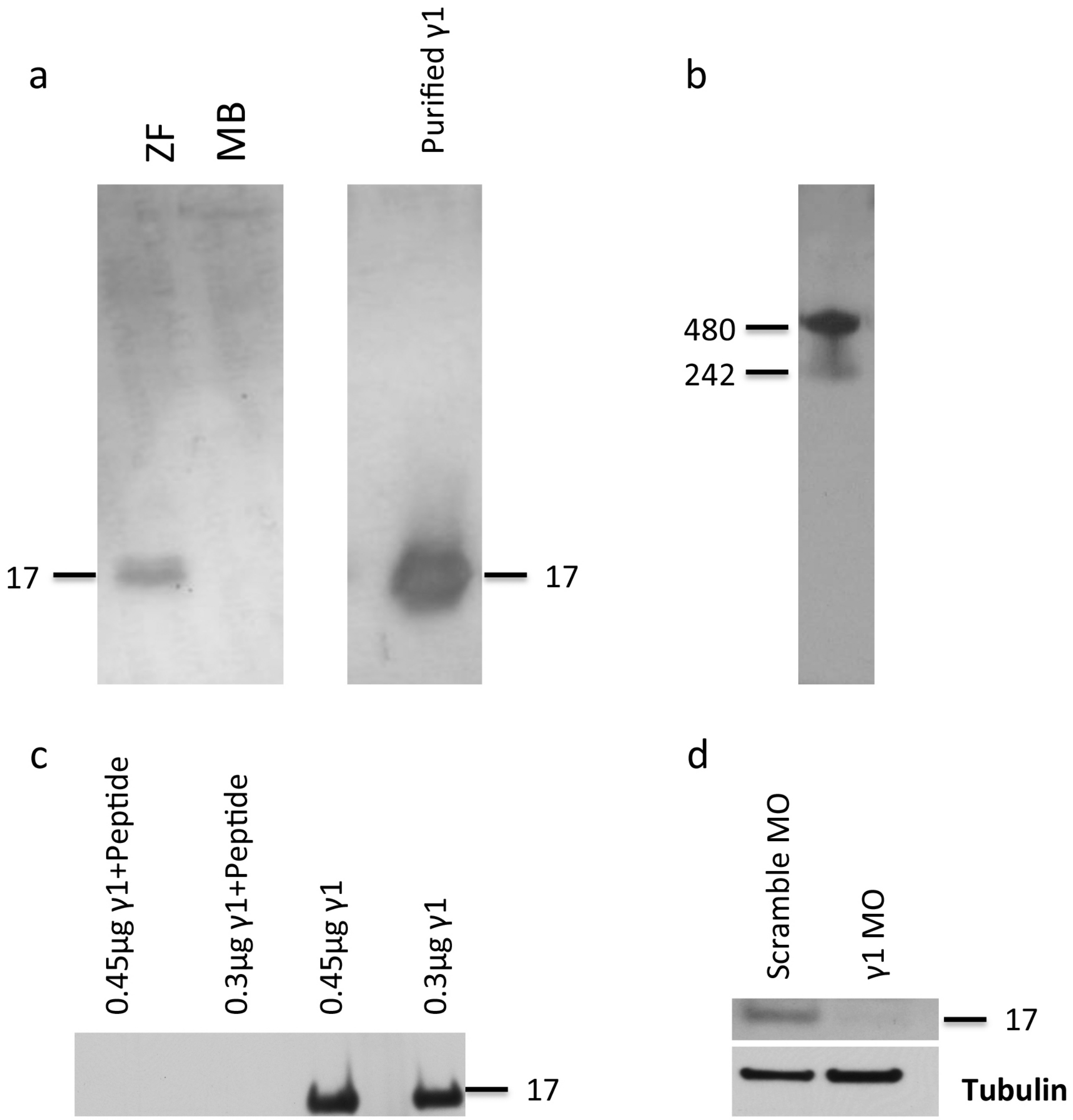

Figure S1: ZF γ1 antibody is specific for ZF. Using denaturing conditions/SDS PAGE, a band for γ1 was detected at 17 kDa for ZF adult brain (ZF) and purified ZF γ1 (a). No ZF γ1 signal was detected for mouse brain (MB). Using non-denaturing conditions/Native PAGE, a major band for ZF γ1 was detected at 480 kDa and a minor band at 242 kDa (b). Peptide preincubated with the ZF γ1 antibody is shown for SDS PAGE. No band for ZF γ1 was detected after preincubation of γ1 antibody with γ1 peptide (c). ZF γ1 MO reduced protein levels of ZF γ1 as compared to scramble MO as determined by SDS PAGE (d).

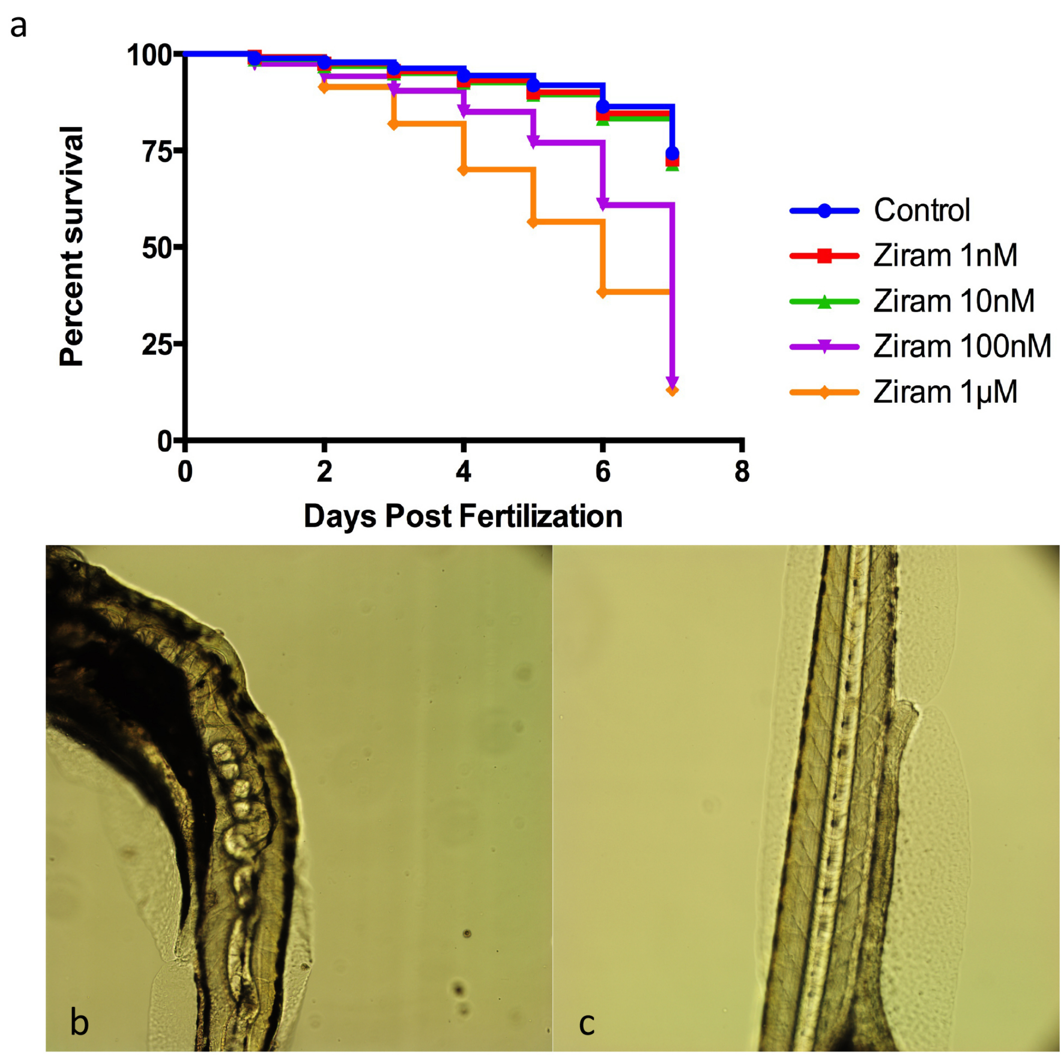

Figure S2: **Ziram is toxic in a concentration-dependent manner and causes notochord distortion.** A significant change in toxicity was observed for embryos treated (24hpf) with 100nM and 1µM ziram by day 7 (n = 50, p<.0001) (a). ZF embryos treated with 50 nM ziram at 5 hpf, were found to have a shorter body axis, pericardial edema, and notochord distortion (b) as compared to vehicle treated ZF (c). p<.0001 Log-Rank test.

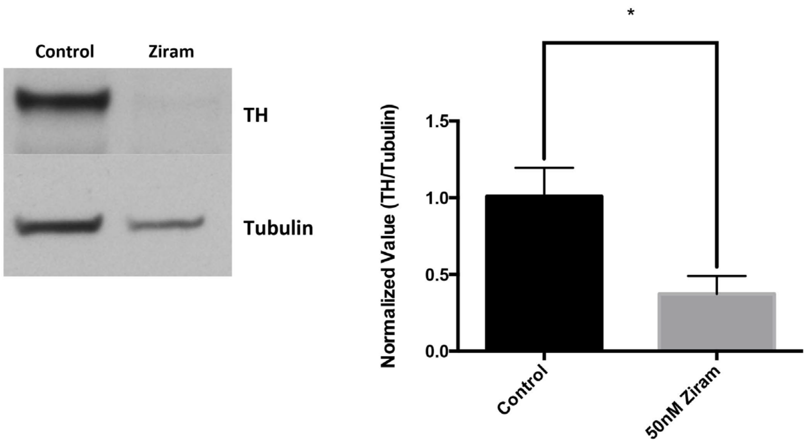

Figure S3. Ziram treatment results in reduction of TH-1 levels. Using Western blot analysis, the effect of ziram (50nM) on TH-1 levels was investigated. A 63% reduction in TH-1 was observed for embryos treated with 50nM ziram vs. controls (n=4; *p-value = .03 using two-tailed T test). Bars represent standard error of the mean.
